# Engineering images designed by fractal subdivision scheme

**DOI:** 10.1186/s40064-016-3018-3

**Published:** 2016-09-06

**Authors:** Ghulam Mustafa, Mehwish Bari, Saba Jamil

**Affiliations:** 1 Department of Mathematics, The Islamia University of Bahawalpur, Baghdad Campus, Bahawalpur, 63100 Punjab Pakistan; 2 National College of Business Administration and Economics, Bahawalpur Campus, Bahawalpur, Punjab Pakistan

**Keywords:** Interpolating subdivision scheme, Engineering images, Fractal antennas, Bearings, 28A80, 65D05, 65D10

## Abstract

This paper is concerned with the modeling of engineering images by the fractal properties of 6-point binary interpolating scheme. Association between the fractal behavior of the limit curve/surface and the parameter is obtained. The relationship between the subdivision parameter and the fractal dimension of the limit fractal curve of subdivision fractal is also presented. Numerical examples and visual demonstrations show that 6-point scheme is good choice for the generation of fractals for the modeling of fractal antennas, bearings, garari’s and rock etc.

## Background

Fractals are infinitely complex patterns that are self-similar across different scales. They are made by rehashing a basic procedure again and again in an ongoing feedback loop. Its uses in various areas of the study of materials and of other areas of engineering are examples of practical prose. Its uses in physical theory, especially in conjunction with the basic equations of mathematical physics. Let us, instead, give a few typical examples: to rock base, an essential of engineering, furthermore a proceeding with objective of science, is to depict nature quantitatively. Another illustration is viscus flow through porous media, i.e. the stream of water pushing oil, has demonstrated as of late to admit to a few administrations, one of which is a ‘front‘, which is a compelling and attractive arrangement and another is ‘fractal fingering‘ which is undesirable.

**Fig. 1:**
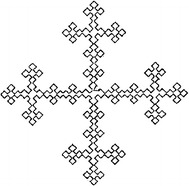
Fractal antenna

A fractal antenna (i.e. Fig. 1) is an antenna that uses a fractal, self-similar design to maximize the length, or increase the perimeter of material that can receive or transmit electromagnetic radiation within a given total surface area or volume. Another mechanical parts in engineering such as gears, chain, grari etc can be constructed based on ideas of fractal geometry.

Owing to the rapid emergence and growth of techniques in the engineering application of fractals, it has become necessary to gather the most recent advances on a regular basis. This study starts from the question, can we design mechanical parts in engineering by simple iterative techniques?

Firstly, Mandelbrot (1982) found fractal geometry that deals with geometric shape which is self-similar, irregular and has detailed structure at arbitrarily small scales. Nowadays, many techniques to generate fractals have been devised, such as IFS (iterated function systems) method Barnsley and Demko (1985), L-system method Prusinkiewicz and Lindenmayer (1990) and few others. Recently it has been shown that subdivision technique is not only an important tool for the fast generation of smooth engineering objects, but also an efficient tool for the fast generation of fractals.

Zheng et al. (2007a, b) analyzed fractal properties of 4-point binary and three point ternary interpolatory subdivision schemes. Wang et al. (2011) discussed the fractal properties of the generalized chaikin corner-cutting subdivision scheme with two tension parameters. They gave the fractal range of scheme on the basis of the discussion of limit points on the limit curve. Li et al. (2013) designed the fractal curves by using the normal vector based subdivision scheme.

Sarfraz et al. (2015) designed some engineering images by using rational spline interpolation. In this paper, we explore the properties of Weissman (1989) fractal subdivision scheme in different areas including engineering images. We conclude that 6-point scheme of Weissman can create engineering images for curves and surfaces with true fractal allotting and can provide some ways of shape control.

The paper is organized as follows. In "Fractal properties of the scheme" section, fractal range of 6-point subdivision scheme is being discussed. In "Numerical examples and demonstrations" section, some numerical examples are presented to confirm the correctness and effectiveness of the engineering images in the form of curve and surface. Finally, we give some concluding remarks in "Conclusions" section.

## Fractal properties of the scheme

The well known 6-point binary interpolating scheme Weissman (1989) is

1 $$\begin{aligned} p_{2i}^{k+2} {} & = & p_i^{k+1}\nonumber \\ p_{2i+1}^{k+2} {} & = \mu p_{i-2}^{k+1}-\left( \frac{1}{16}+3\mu \right) p_{i-1}^{k+1}+\left( \frac{9}{16}+2\mu \right) p_{i}^{k+1}+\left( \frac{9}{16}+2\mu \right) p_{i+1}^{k+1}\nonumber \\&-\left( \frac{1}{16}+3\mu \right) p_{i+2}^{k+1}+\mu p_{i+3}^{k+1}. \end{aligned}$$

The scheme (1) produces $C^0$- and $C^1$- continuous curves at $-\frac{3}{16}<\mu <\frac{-1+3\sqrt{2}}{16}$ and $\frac{-6+3\sqrt{2}}{32}<\mu <\frac{-1+\sqrt{19}}{32}$ respectively. By substituting $\mu =0$ and $\mu =\frac{3}{256}$, we get 4-point and 6-point schemes as given in Deslauriers and Dubuc (1989) respectively.

According to interpolatory property $p_0^k\equiv p_0^0$, $k\ge 0$. Suppose $p_i^m$ and $p_j^m$ are two fixed control points after *m* subdivision steps, $\forall \, m\in \mathbb {Z}, m\ge 0$. The role of parameter $\mu$ is required to be evaluated on the sum of all small edges among the two points after another *k* iterations. First we discuss and analyze the effect of $\mu$ among the two initial control points $p_0^0$ and $p_1^0$.

For $i=-2$ in the odd rule $p^{k+1}_{2i+1}$ of scheme (1), we have

2 $$\begin{aligned} p_{-3}^{k+2} & = \mu \left( p_{-4}^{k+1}+p_{1}^{k+1}\right) -\left( \frac{1}{16}+3\mu \right) \left( p_{-3}^{k+1}+p_0^{k+1}\right) +\left( \frac{9}{16}+2\mu \right) \left( p_{-2}^{k+1}+p_{-1}^{k+1}\right) .\nonumber \\ \end{aligned}$$

Substituting $p_{-4}^{k+1}=p_{-2}^{k}$ and $p_{-2}^{k+1}=p_{-1}^{k}$ in (2), we get

$$\begin{aligned} p_{-3}^{k+2} & = \mu \left( p_{-2}^{k}+p_{1}^{k+1}\right) -\left( \frac{1}{16}+3\mu \right) \left( p_{-3}^{k+1}+p_0^{0}\right) +\left( \frac{9}{16}+2\mu \right) \left( p_{-1}^{k}+p_{-1}^{k+1}\right) .\\ \end{aligned}$$

Putting $i=-1,0$ in odd rule $p^{k+1}_{2i+1}$ of scheme (1) by using $p_{-2}^{k+1}=p_{-1}^k$ and $p_{2}^{k+1}=p_1^k$, we have

$$\begin{aligned} p_{-1}^{k+2} & = \mu \left( p_{-3}^{k+1}+p_{1}^{k}\right) -\left( \frac{1}{16}+3\mu \right) \left( p_{-1}^{k}+p_1^{k+1}\right) +\left( \frac{9}{16}+2\mu \right) \left( p_{-1}^{k+1}+p_{0}^{0}\right) ,\\ \end{aligned}$$

and

$$\begin{aligned} p_{1}^{k+2} & = \mu \left( p_{-1}^{k}+p_{3}^{k+1}\right) -\left( \frac{1}{16}+3\mu \right) \left( p_{-1}^{k+1}+p_1^{k}\right) +\left( \frac{9}{16}+2\mu \right) \left( p_{1}^{k+1}+p_{0}^{0}\right) . \end{aligned}$$

Here, we define two edge vectors between the points $p_0^0$ and $p_1^0$ after *k* steps defined by $v_k=p_1^k-p_0^k$ and $R_k=p_2^k-p_1^k$. Since $p_i^k=\frac{i}{2^k}$ so we have $p_2^k=\frac{2}{2^k}=\frac{1}{2^{k-1}}=p_1^{k-1}$ and we can write as

3 $$\begin{aligned} R_k=p_1^{k-1}-p_0^{k-1}+p_0^k-p_1^k=v_{k-1}-v_k. \end{aligned}$$

Let $U_k=p_1^k-p_{-1}^k$, $W_k=p_0^k-p_{-1}^k$, we can write it as $U_k-W_k=p_1^k-p_{-1}^k-p_0^k+p_{-1}^k = p_1^k-p_0^k=v_k.$ Further $U_k=v_k+W_k$. This implies

$$\begin{aligned} U_{k+2} & = -\mu \left( p_1^k-p_{-1}^k\right) +\mu \left( p_3^{k+1}-p_{-3}^{k+1}\right) -\left( \frac{1}{16}+3\mu \right) \left( p_1^{k}-p_{-1}^{k}\right) \\&\quad + \left( \frac{1}{16}+3\mu \right) \left( p_1^{k+1}-p_{-1}^{k+1}\right) +\left( \frac{9}{16}+2\mu \right) \left( p_1^{k+1}-p_{-1}^{k+1}\right) . \end{aligned}$$

Since by dyadic parametrization $p_{i}^{k+1}= \frac{i}{2^{k+1}}$ then the term $\mu \left( p_3^{k+1}-p_{-3}^{k+1}\right)$ can be written as

$$\begin{aligned} \mu \left( \frac{1}{2^{k+1}} +\frac{2}{2^{k+1}} -\frac{-2}{2^{k+1}} -\frac{-1}{2^{k+1}} \right) =\mu \left( \frac{1}{2^{k+1}} -\frac{-1}{2^{k+1}} +\frac{1}{2^{k}} -\frac{-1}{2^{k}} \right) . \end{aligned}$$

Therefore

$$\begin{aligned} U_{k+2} & = -\mu \left( p_{1}^{k}-p_{-1}^{k}\right) +\mu \left( p_{1}^{k+1}-p_{-1}^{k+1}+p_1^{k}-p_{-1}^{k}\right) - \left( \frac{1}{16}+3\mu \right) \\&\quad \times \left( p_{1}^{k}-p_{-1}^{k}\right) +\left( \frac{1}{16}+3\mu \right) \left( p_{1}^{k+1}-p_{-1}^{k+1}\right) +\left( \frac{9}{16}+2\mu \right) \left( p_{1}^{k+1}-p_{-1}^{k+1}\right) . \end{aligned}$$

This implies

$$\begin{aligned} U_{k+2}=\mu U_{k+1}-\left( \frac{1}{16}+3\mu \right) U_k+\left( \frac{1}{16}+3\mu \right) U_{k+1}+\left( \frac{9}{16}+2\mu \right) U_{k+1}. \end{aligned}$$

So we have

4 $$\begin{aligned} U_{k+2}=\left( \frac{10}{16}+6\mu \right) U_{k+1}-\left( \frac{1}{16}+3\mu \right) U_k. \end{aligned}$$

Equation (4) is the second order linear difference equation, such that

$$\begin{aligned} U_{k+2}-\left( \frac{10}{16}+6\mu \right) U_{k+1}+\left( \frac{1}{16}+3\mu \right) U_k=0. \end{aligned}$$

The characteristic equation is

5 $$\begin{aligned} Q^2-\left( \frac{10}{16}+6\mu \right) Q+\left( \frac{1}{16}+3\mu \right) =0. \end{aligned}$$

By solving (5), we get $Q_1=\frac{1}{8}+6\mu$ and $Q_2=\frac{1}{2}$, $\left( Q_1\ne Q_2\right)$ when $\mu \ne \frac{1}{16}$.

Let $U_0=p_1^0-p_{-1}^0$ and $U_1=p_1^1-p_{-1}^1$. For $k+1=0$ and $i=0$ in (1), we get

$$\begin{aligned} p_1^1 & = \mu p_{-2}^0-\left( \frac{1}{16}+3\mu \right) p_{-1}^0+\left( \frac{9}{16}+2\mu \right) p_0^0+\left( \frac{9}{16}+2\mu \right) p_1^0 \\&-\left( \frac{1}{16}+3\mu \right) p_{2}^0+\mu p_{3}^0. \end{aligned}$$

This implies

6 $$\begin{aligned} p_1^1=\mu \left( p_{-2}^0+p_3^0\right) -\left( \frac{1}{16}+3\mu \right) \left( p_{-1}^0+p_2^0\right) +\left( \frac{9}{16}+2\mu \right) \left( p_0^0+p_1^0\right) . \end{aligned}$$

Substitute $k+1=0$ and $i=-1$ in (1), we have

$$\begin{aligned} p_{-1}^1 &= \mu p_{-3}^0-\left( \frac{1}{16}+3\mu \right) p_{-2}^0+\left( \frac{9}{16}+2\mu \right) p_{-1}^0+\left( \frac{9}{16}+2\mu \right) p_0^0 \\&-\left( \frac{1}{16}+3\mu \right) p_{1}^0+\mu p_{2}^0. \end{aligned}$$

This implies

7 $$\begin{aligned} p_{-1}^1=\mu \left( p_{-3}^0+p_2^0\right) -\left( \frac{1}{16}+3\mu \right) \left( p_{-2}^0+p_1^0\right) +\left( \frac{9}{16}+2\mu \right) \left( p_{-1}^0+p_0^0\right) . \end{aligned}$$

Subtracting (7) from (6), we have

8 $$\begin{aligned} p_{1}^1-p_{-1}^1 &= -\mu p_{-3}^0+\left( \frac{1}{16}+4\mu \right) p_{-2}^0-\left( \frac{5}{8}+5\mu \right) p_{-1}^0+\left( \frac{5}{8}+5\mu \right) p_1^0\nonumber \\&- \left( \frac{1}{16}+4\mu \right) p_{2}^0+\mu p_{3}^0. \end{aligned}$$

The solution of Eq. (8) is

$$\begin{aligned} U_k &= \left( \frac{1}{8}+6\mu \right) ^kc_1+\left( \frac{1}{2}\right) ^kc_2, \end{aligned}$$

where

$$\begin{aligned} c_1 &= \frac{8}{3-48\mu }\left\{ \mu p_{-3}^0-\left( \frac{1}{16}+4\mu \right) p_{-2}^0+\left( \frac{1}{8}+5\mu \right) p_{-1}^0- \left( \frac{1}{8}+5\mu \right) p_1^0 \right. \\&\left. +\left( \frac{1}{16}+4\mu \right) p_{2}^0-\mu p_3^0\right\} \end{aligned}$$

and

$$\begin{aligned} c_2 &= -\frac{8}{3-48\mu }\left\{ \mu p_{-3}^0-\left( \frac{1}{16}+4\mu \right) p_{-2}^0+\left( \frac{1}{2}-\mu \right) p_{-1}^0- \left( \frac{1}{2}-\mu \right) p_1^0 \right. \\&\left. +\left( \frac{1}{16}+4\mu \right) p_{2}^0-\mu p_3^0\right\} . \end{aligned}$$

When $\mu =\frac{1}{16}$, the solution of Eq. (4) will be

$$\begin{aligned} U_k=\left( \frac{1}{2}\right) ^k\acute{c}_1+\left( \frac{1}{2}\right) ^{k}k\acute{c}_2 \end{aligned}$$

where

$$\begin{aligned} \acute{c}_1=p_1^0-p_{-1}^0 \end{aligned}$$

and

$$\begin{aligned} \acute{c}_2=-\frac{1}{8}\left( p_{-3}^0-5p_{-2}^0+7p_{-1}^0-7p_{1}^0+5p_{2}^0-p_{3}^0\right) . \end{aligned}$$

Since

$$\begin{aligned} v_{k+2} &= \mu \left( p_{-2}^{k+1}+p_3^{k+1}\right) -\left( \frac{9}{16}-\frac{8}{16}+2\mu +\mu \right) \left( p_{-1}^{k+1}+p_2^{k+1}\right) \\&+ \left( \frac{9}{16}+2\mu \right) \left( p_{0}^{k+1}+p_1^{k+1}\right) -p_0^{k+2}. \end{aligned}$$

Then by taking $U_{k+1}=p_1^{k+1}-p_{-1}^{k+1}$ and $v_k=p_1^{k}-p_0^k$, we get

$$\begin{aligned} v_{k+2} &= \left( \frac{9}{16}+2\mu \right) U_{k+1}-\left( \frac{9}{16}+2\mu \right) v_k-\mu \left( p_{2}^{k+1}-p_{-2}^{k+1}\right) +\mu \left( p_3^{k+1}-p_{-1}^{k+1}\right) \\&+\frac{1}{2}p_{-1}^{k+1}+\frac{1}{2}p_2^{k+1}-p_0^{k+2}. \end{aligned}$$

Since by dyadic parametrization $-\mu \left( p_{2}^{k+1}-p_{-2}^{k+1}\right) +\mu \left( p_3^{k+1}-p_{-1}^{k+1}\right) =0$ then

$$\begin{aligned} v_{k+2}=\left( \frac{9}{16}+2\mu \right) U_{k+1}-\left( \frac{9}{16}+2\mu \right) v_{k}+\frac{1}{2}\left( p_{1}^{k}-p_0^k\right) +\frac{1}{2}\left( p_{-1}^{k+1}-p_0^{k+1}\right) . \end{aligned}$$

Since $v_{k}=p_{1}^{k}-p_0^k$ and $W_{k+1}=p_{0}^{k}-p_{-1}^k$. Then

9 $$\begin{aligned} v_{k+2}=\left( \frac{9}{16}+2\mu \right) U_{k+1}-\left( \frac{9}{16}+2\mu \right) v_{k}+\frac{1}{2}v_k-\frac{1}{2}W_{k+1}. \end{aligned}$$

Substitute $W_{k+1}=U_{k+1}-v_{k+1}$ in (9), we get

$$\begin{aligned} v_{k+2}=\left( \frac{9}{16}+2\mu \right) U_{k+1}-\left( \frac{9}{16}+2\mu \right) v_{k}+\frac{1}{2}v_k-\frac{1}{2}\left( U_{k+1}-v_{k+1}\right) . \end{aligned}$$

Simplifying, we have

10 $$\begin{aligned} v_{k+2}-\frac{1}{2}v_{k+1}+\left( \frac{1}{16}+2\mu \right) v_{k}=\left( \frac{1}{16}+2\mu \right) U_{k+1}. \end{aligned}$$

Now we will find the solution of Eq. (10), first consider

11 $$\begin{aligned} \gamma ^2-\frac{1}{2}\gamma +\left( \frac{1}{16}+2\mu \right) =0. \end{aligned}$$

**Case 1**. When $-\frac{3}{16}<\mu <\frac{-6+3\sqrt{2}}{32}$, the solution of Eq. (11) is $\gamma _1=\frac{1}{4}+\sqrt{-2\mu }$ and $\gamma _2=\frac{1}{4}-\sqrt{-2\mu }$, and $\gamma _1\ne \gamma _2$, $\gamma _{1}, \gamma _{2}\ne \frac{1}{8}+6\mu$, $\gamma _{1}, \gamma _{2}\ne \frac{1}{2}$. Equation (10) can be expressed as

$$\begin{aligned} v_{k+2}-\frac{1}{2}v_{k+1}+\left( \frac{1}{16}+2\mu \right) v_{k}=\left( \frac{1}{16}+2\mu \right) \left\{ c_1\left( \frac{1}{8}+6\mu \right) ^{k+1}+c_2\left( \frac{1}{2}\right) ^{k+1}\right\} . \end{aligned}$$

The solution of above equation is

12 $$\begin{aligned} v_{k}=\beta _1\gamma _1^k+\beta _2\gamma _2^k+\frac{65}{144}c_1\left( \frac{1}{8}+6\mu \right) ^k+\frac{1}{2}c_2\left( \frac{1}{2}\right) ^k, \end{aligned}$$

where $\beta _1$ and $\beta _2$ are given as

$$\begin{aligned} \beta _1 =& {\frac{1}{13824{\sqrt{-2\,w} \left( -1+16\,w \right) }}}\left\{ -3568\,\sqrt{-2\,w}p_{{-1}}+6912\,p_{{0}} \sqrt{-2\,w} -56\,\sqrt{-2\,w}p_{{2}}\right. \\&\left. +\,56\,\sqrt{-2\,w}p_{{-2}}-3344 \,p_{{1}}\sqrt{-2\,w}+896\,\sqrt{-2\,w}wp_{{3}}+59776\,p_{{1}}\sqrt{-2\,w}w\right. \\&\left. -\,110592\,p_{{0}}\sqrt{-2\,w}w+50816\,\sqrt{-2\,w}p_{{-1}}w- 896\,\sqrt{-2\,w}wp_{{-3}}-3584\,\sqrt{-2\,w}p_{{2}}w\right. \\&\left. +\,3584\,\sqrt{- 2\,w}wp_{{-2}}+1296\,p_{{0}}-878\,p_{{1}}+209\,p_{{-2}}+223\,p_{{2}}- 850\,p_{{-1}}-3344\,wp_{{-3}}\right. \\&\left. 
-\,3568\,wp_{{3}}+3344\,wp_{{-2}}-28416\,p_ {{1}}{w}^{2}+3568\,p_{{2}}w+221184\,p_{{0}}{w}^{2}+60672\,{w}^{2}p_{{3 }}\right. \\&\left. -\,89088\,{w}^{2}p_{{-2}}-34560\,p_{{0}}w+17392\,p_{{1}}w+49920\,{w}^{ 2}p_{{-3}}+17168\,p_{{-1}}w\right. \\&\left. -\,132096\,p_{{2}}{w}^{2}-82176\,p_{{-1}}{w}^ {2}\right\} ,\\ \beta _2 &= {\frac{1}{13824\sqrt{-2\,w} \left( -1+16\,w \right) }}\,\left\{ -209\,p_{{-2}}-3344\,p_{{1}}\sqrt{-2\,w}- 56\,\sqrt{-2\,w}p_{{2}}\right. \\&\left. +\,6912\,p_{{0}}\sqrt{-2\,w} -1296\,p_{{0}}+878 \,p_{{1}}-223\,p_{{2}}-17392\,p_{{1}}w-49920\,{w}^{2}p_{{-3}}\right. \\&\left. +\,89088\,{ w}^{2}p_{{-2}}+82176\,p_{{-1}}{w}^{2}+28416\,p_{{1}}{w}^{2}+132096\,p_ {{2}}{w}^{2}-60672\,{w}^{2}p_{{3}}\right. \\&\left. -\,221184\,p_{{0}}{w}^{2}-896\,\sqrt{ -2\,w}wp_{{-3}}-3568\,\sqrt{-2\,w}p_{{-1}}+56\,\sqrt{-2\,w}p_{{-2}}\right. \\&\left. -\,110592\,p_{{0}}\sqrt{-2\,w}w+50816\,\sqrt{-2\,w}p_{{-1}}w+59776\,p_{ {1}}\sqrt{-2\,w}w\right. \\&\left. +\,896\,\sqrt{-2\,w}wp_{{3}}-3584\,\sqrt{-2\,w}p_{{2 }}w+3584\,\sqrt{-2\,w}wp_{{-2}}+34560\,p_{{0}}w\right. \\&\left. -\,3344\,wp_{{-2}}+3568 \,wp_{{3}}+3344\,wp_{{-3}}-17168\,p_{{-1}}w-3568\,p_{{2}}w+850\,p_{{-1 }}\right\} . \end{aligned}$$

From Eqs. (3) and (12), we have

13 $$\begin{aligned} R_k =& \left( \frac{1}{\gamma _1}-1\right) \beta _1\gamma _1^k+\left( \frac{1}{\gamma _2}-1\right) \beta _2\gamma _2^k+\left( \frac{7-48\mu }{1+48\mu }\right) \frac{65}{144} c_1\left( \frac{1}{8}+6\mu \right) ^k \nonumber \\&+\frac{1}{2}c_2\left( \frac{1}{2}\right) ^k. \end{aligned}$$

**Case 2.** When $\frac{-1+\sqrt{19}}{32}<\mu <\frac{-1+3\sqrt{2}}{16}$, the solution of Eq. (11) is $\rho _1=\frac{1}{4}+i\sqrt{2\mu }$ and $\rho _2=\frac{1}{4}-i\sqrt{2\mu }$, $\rho _1\ne \rho _2$, $\rho _{1}, \rho _{2}\ne \frac{1}{8}+6\mu$, $\rho _{1}, \rho _{2}\ne \frac{1}{2}$. The solution of Eq. (10) can be expressed as

14 $$\begin{aligned} v_{k}={\chi }_1\rho _1^k+{\chi }_2\rho _2^k+\frac{65}{144}c_1\left( \frac{1}{8}+6\mu \right) ^k+\frac{1}{2}c_2\left( \frac{1}{2}\right) ^k, \end{aligned}$$

where ${\chi }_1$ and ${\chi }_2$ are given as

$$\begin{aligned} {\chi }_1 =& {\frac{1}{13824i{\sqrt{2\,w} \left( -1+16\,w \right) }}}\left\{ -3568i\,\sqrt{2\,w}p_{{-1}}+6912i\,p_{{0}} \sqrt{2\,w} -56i\,\sqrt{2\,w}p_{{2}}\right. \\&\left. +\,56i\,\sqrt{-2\,w}p_{{2}}-3344i \,p_{{1}}\sqrt{2\,w}+896i\,\sqrt{2\,w}wp_{{3}}+59776i\,p_{{1}}\sqrt{2\,w}w\right. \\&\left. -\,110592i\,p_{{0}}\sqrt{2\,w}w+50816i\,\sqrt{2\,w}p_{{-1}}w- 896i\,\sqrt{2\,w}wp_{{-3}}-3584i\,\sqrt{2\,w}p_{{2}}w\right. \\&\left. +\,3584i\,\sqrt{- 2\,w}wp_{{-2}}+1296\,p_{{0}}-878\,p_{{1}}+209\,p_{{-2}}+223\,p_{{2}}- 850\,p_{{-1}}-3344\,wp_{{-3}}\right. \\&\left. -\,3568\,wp_{{3}}+3344\,wp_{{-2}}-28416\,p_ {{1}}{w}^{2}+3568\,p_{{2}}w+221184\,p_{{0}}{w}^{2}+60672\,{w}^{2}p_{{3 }}\right. \\&\left. -\,89088\,{w}^{2}p_{{-2}}-34560\,p_{{0}}w+17392\,p_{{1}}w+49920\,{w}^{ 2}p_{{-3}}+17168\,p_{{-1}}w\right. \\&\left. -\,132096\,p_{{2}}{w}^{2}-82176\,p_{{-1}}{w}^ {2}\right\} ,\\ {\chi }_2 =& {\frac{1}{13824i\sqrt{2\,w} \left( -1+16\,w \right) }}\,\left\{ -209\,p_{{-2}}-3344i\,p_{{1}}\sqrt{2\,w}- 56i\,\sqrt{2\,w}p_{{2}}\right. \\&\left. +\,6912i\,p_{{0}}\sqrt{2\,w} -1296\,p_{{0}}+878 \,p_{{1}}-223\,p_{{2}}-17392\,p_{{1}}w-49920\,{w}^{2}p_{{-3}}\right. \\&\left. +\,89088\,{ w}^{2}p_{{-2}}+82176\,p_{{-1}}{w}^{2}+28416\,p_{{1}}{w}^{2}+132096\,p_ {{2}}{w}^{2}-60672\,{w}^{2}p_{{3}}\right. \\&\left. -\,221184\,p_{{0}}{w}^{2}-896i\,\sqrt{ 2\,w}wp_{{-3}}-3568i\,\sqrt{2\,w}p_{{-1}}+56i\,\sqrt{2\,w}p_{{-2}}\right. \\&\left. -\,110592i\,p_{{0}}\sqrt{2\,w}w+50816i\,\sqrt{2\,w}p_{{-1}}w+59776i\,p_{ {1}}\sqrt{2\,w}w\right. \\&\left. +\,896i\,\sqrt{2\,w}wp_{{3}}-3584i\,\sqrt{2\,w}p_{{2 }}w+3584i\,\sqrt{2\,w}wp_{{-2}}+34560\,p_{{0}}w\right. \\&\left. -\,3344\,wp_{{-2}}+3568 \,wp_{{3}}+3344\,wp_{{-3}}-17168\,p_{{-1}}w-3568\,p_{{2}}w+850\,p_{{-1 }}\right\} . \end{aligned}$$

From Eqs. (3) and (14), we have

15 $$\begin{aligned} R_k &= \left( \frac{1}{\rho _1}-1\right) {\chi }_1\rho _1^k+\left( \frac{1}{\rho _2}-1\right) {\chi }_2\rho _2^k+\left( \frac{7-48\mu }{1+48\mu }{} \right) \frac{65}{144} c_1\left( \frac{1}{8}+6\mu \right) ^k \nonumber \\&+\frac{1}{2}c_2\left( \frac{1}{2}\right) ^k. \end{aligned}$$

From the Eqs. (12)–(15), we have the following theorems.

### **Theorem 1**

*For*$\frac{-1+\sqrt{19}}{32}<\mu <\frac{-1+3\sqrt{2}}{16}$, *the limit curve of the 6-point scheme is a fractal curve.*

### *Proof*

By following the Eqs. (14) and (15), we may conclude that the $2^k$ small edge vectors between $p_0^0$ and $p_1^0$ after *k* subdivision steps can be expressed by induction as:

16 $$\begin{aligned} E_j^k=p_j^k-p_{j-1}^k=\varsigma _{1j}\rho _1^k+\varsigma _{2j}\rho _2^k+\varsigma _{3j}\left( \frac{1}{8}+6\mu \right) ^k+\varsigma _{4j}\left( \frac{1}{2}\right) ^k, \end{aligned}$$

for $j=1, 2,\,\ldots\,,2^k$, where $\rho _{ij}\ne 0$, $i=1,2,3,4.$ The Eq. (16) is written by the linear combination of (14) and (15).

By taking $\xi =\frac{1}{8}+6\mu$ and $\xi >|\rho _1|, |\rho _2|$. Let $\mid v\mid$ denote the length of a vector *v* and $\mid E_{j_0}^k\mid =min_{j=1,...,2^k} \mid E_{j_0}^k\mid$, then we get

$$\begin{aligned} \sum _{j=1}^{2^k}|E_j^k|\ge 2^k|E_{j_0}^k|=2^k\left| \varsigma _{1j_0}\rho _1^k+\varsigma _{2j_0}\rho _2^k+\varsigma _{3j_0}\xi ^k+\varsigma _{4j_0}\left( \frac{1}{2}\right) ^k\right| . \end{aligned}$$

This implies

$$\begin{aligned} \sum _{j=1}^{2^k}|E_j^k| =\left( 2\xi \right) ^k\left| \varsigma _{1j_0}\left( \frac{\rho _1}{\xi }\right) ^k+\varsigma _{2j_0}\left( \frac{\rho _2}{\xi }\right) ^k+\varsigma _{3j_0}+\varsigma _{4j_0}\left( \frac{1}{2\xi }\right) ^k\right| \rightarrow \infty \, as\, k\rightarrow \infty . \end{aligned}$$

The sum of the length of all the small edges between $p_0^0$ and $p_1^0$ after *k* subdivision steps grows without bound when *k* approaches to infinity, so by Zheng et al. (2007a, (2007b) the limit curve of 6-point scheme is a fractal curve in the range $\frac{-1+\sqrt{19}}{32}<\mu <\frac{-1+3\sqrt{2}}{16}$. $\square$

Similarly, we get following theorem.

### **Theorem 2**

*For*$-\frac{3}{16}<\mu <\frac{-6+3\sqrt{2}}{32}$, *the limit curve of the 6-point scheme is a fractal curve.*

A straightforward generalization of 6-point scheme is its tensor-product version which generates fractal surfaces over the parametric ranges $-\frac{3}{16}<\mu <\frac{-6+3\sqrt{2}}{32}$ and $\frac{-1+\sqrt{19}}{32}<\mu <\frac{-1+3\sqrt{2}}{16}$.

### Complexity of subdivision fractal

We measure the complexity of the 6-point binary interpolating subdivision fractal by estimating its box dimension. By using Theorem 1 and adopting the procedures of Zheng et al. (2010), Wang and Qian (2003), we get following theorem.

#### **Theorem 3**

*If*$p^k_i$*are the control points at levelkand*$f_i^k=\left( 2^k\right) ^\alpha \left( p^k_{i+1}-p^k_i\right)$*then the sequence*$\mid f_i^k\mid$*is bounded above, where*$\alpha =1-\log _{l}^{32\mu }$, $l=-1+\sqrt{19}$*and*$\frac{-1+\sqrt{19}}{32}<\mu <\frac{-1+3\sqrt{2}}{16}$.

By using Theorem 3 and taking up the methods of Zheng et al. (2010), Wang and Qian (2003), we get following theorem.

#### **Theorem 4**

*The fractal dimension of the 6-point binary interpolatory subdivision fractal is*$d=2-\alpha \cong 2.5430$.

From Theorem 1, we know that subdivision fractal can be gotten by keeping the corresponding subdivision parameter $\mu$ within the interval $\frac{-1+\sqrt{19}}{32}<\mu <\frac{-1+3\sqrt{2}}{16}$. Therefore by Theorem 4 the fractal dimension of the 6-point subdivision fractal as a limit will be no more than $d=2-\alpha \cong 2.5430$. Similarly, one can compute the fractal dimension of subdivision fractal for the interval $-\frac{3}{16}<\mu <\frac{-6+3\sqrt{2}}{32}$.

## Numerical examples and demonstrations

**Fig. 2:**
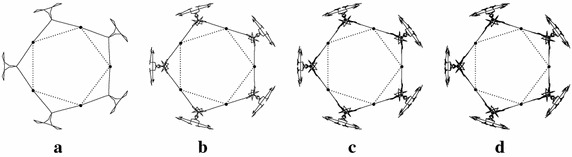
Fractals: *Dotted lines* with initial control points show the initial control polygon (**a**, **b**, **c**, **d**) whereas *solid lines* show the fractal antennas at third, seventh, tenth and thirteenth level with $\mu =0.1718$ respectively

The proposed work is used to construct the engineering structures such as fractal antennas, bearings and garari’s etc. Figure 2a–d present the fractal antennas generated after third, seventh, tenth and thirteenth subdivision levels at $\mu =0.1718$. The fractal dimension of these fractals is 2.4066.

**Fig. 3:**
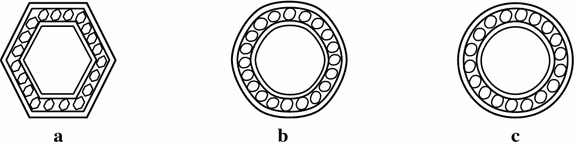
Smooth curves: **a** shows the initial structure of bearing and **b**, **c** shows the bearing at third level with $\mu =0$ and $\mu =\frac{3}{256}$ respectively

**Fig. 4:**
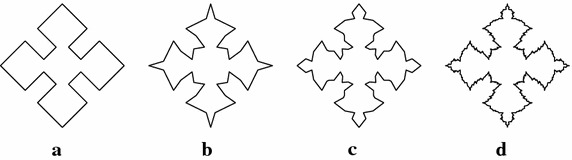
Fractals: **a** shows the initial structure of fractal antenna and **b**–**d** show the fractal antennas at first, second and fourth iteration of the scheme (1) at parametric value $\mu =-0.099$ respectively

**Fig. 5:**
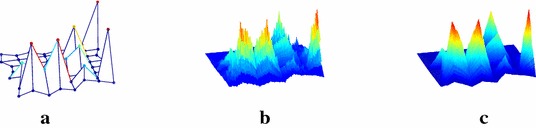
Fractal surfaces: **a** shows the initial control polygon whereas **b**, **c** show the rock surfaces at third level with $\mu =-0.1$ and $\mu =-0.05$ respectively

**Fig. 6:**
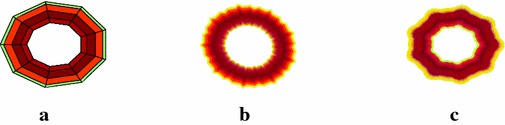
Fractal surfaces: **a** shows the initial structure of garari and **b**, **c** show the garari at third level with $\mu =-0.187$ and $-0.08$ respectively

The initial sample of another fractal antenna is shown in Fig. 3a. Figure 3b, c show the fractal antennas generated by the scheme (1) at $\mu =-0.099$. Figure 4a shows the initial sample for a bearing and Fig. 4b, c show the actual bearing generated at parametric values $\mu =0$ and $\mu =\frac{3}{256}$ respectively. The initial mesh for rock surface is shown in Fig. 5a. Figure 5b, c show the rock surfaces at third level with $\mu =-0.1$ and $\mu =-0.05$ respectively. Figure 6 presents the structure of garari type shapes.

In the case of given initial control points, shapes and dimensions of the fractals can be adjusted and controlled by adjusting the parameter $\mu$. Hence the obtained results in "Fractal properties of the scheme" section can be used to generate fractal in a fast and efficient way.

## Conclusions

In this article, we have reorganized the engineering images by fractal subdivision scheme. We have identified two different parametric intervals to generate different types of engineering models. The relationship between the subdivision parameter and the fractal dimension of the limit fractal curve of the 6-point binary interpolatory subdivision fractal is also presented. It is concluded that 6-point subdivision scheme is an efficient tool for the fast generation of self similar fractals useful in fractal antennas. It is also an appropriate technique for the designing of bearings and garari’s etc.
